# Characterization and Crystal Structural Analysis of Novel Carvedilol Adipate and Succinate Ethanol-Solvated Salts

**DOI:** 10.3390/molecules29194704

**Published:** 2024-10-04

**Authors:** Li Ye, Takayuki Furuishi, Takefumi Yamashita, Etsuo Yonemochi

**Affiliations:** 1 Department of Physical Chemistry, School of Pharmacy and Pharmaceutical Sciences, Hoshi University, 2-4-41 Ebara, Shinagawa-ku 142-8501, Tokyo, Japanyamashita.takefumi@hoshi.ac.jp (T.Y.); 2 Juntendo University Faculty of Pharmacy, 6-8-1 Hinode, Urayasu 279-0013, Chiba, Japan; 3 School of Pharmacy at Narita, International University of Health and Welfare, 4-3 Kozunomori, Narita 286-8686, Chiba, Japan

**Keywords:** carvedilol, adipic acid, succinic acid, crystal structure, solubility, morphology, attachment energy

## Abstract

Two ethanol-solvated adipate and succinate salts of carvedilol (CVD), a Biopharmaceutics Classification System class 2 drug, were synthesized by crystallizing ethanol with adipic acid (ADP) and succinic acid (SUA). Proton transfer from ADP and SUA to CVD and the presence of ethanol in the two novel compounds were confirmed using powder X-ray diffraction, Fourier transform infrared spectroscopy, differential scanning calorimetry, thermogravimetric analysis, and single-crystal X-ray diffraction measurements. The two novel ethanol-solvated salts exhibited enhanced solubility and dissolution rates compared with pure carvedilol in phosphate buffer (pH 6.8). Additionally, the morphologies and attachment energies of the two novel compounds and pure CVD were calculated based on their single-crystal structures, revealing a correlation between attachment energy and dissolution rate.

## 1. Introduction

Solid-state chemistry encompasses the synthesis, structure, and physicochemical properties of materials. Classification of the solid state is straightforward and can be divided into amorphous and crystalline forms. Crystalline solids can be further divided into single-component and multi-component systems. Multi-component systems can be categorized as salts or co-crystals based on the presence of ionic bonds and are further classified as solvates or hydrates depending on whether they contain solvent molecules or water [1,2,3,4,5]. Additionally, an API can form polymorphs, that is, different crystals of the same chemical entity. However, an API can also form different crystal structures with solvent molecules, i.e., solvates [6]. Solvent molecules often form hydrogen bonds and coordinate covalent bonds with the APIs or excipients in the crystal lattice. Most low-molecular-weight APIs and excipients readily form solvates because of their small molecular sizes. Some solvent molecules, such as water and alcohols, have both hydrogen bond donor and acceptor atoms that can form intermolecular hydrogen bonds with host molecules. The presence of solvent molecules affects the degree of intermolecular interactions (enthalpy) and the degree of crystalline disorder (entropy). Hence, it affects the free energy, thermodynamic parameters, solubility, dissolution rate, solid-state stability, and bioavailability of the solvated APIs. The mechanical properties and deformation mechanisms of a drug product, such as tableting and grinding/milling, can also vary among the different solid forms [7].

According to the Biopharmaceutics Classification System (BCS), over 50% of drug candidates belong to class 2, and over 25% belong to class 4; therefore, the major challenge for over 75% of all drug development candidates is enhancing their solubility [8]. The solubility and dissolution rate of a drug are key factors that determine its efficacy. Over the last three decades, the designing and synthesizing of novel multi-component pharmaceutical co-crystals and drugs has become a common strategy to improve the physical properties of drugs, especially in terms of their dissolution, solubility, and stability [9,10,11].

Historically, pharmaceutical salts have been the first choice for overcoming the problems of poor solubility and dissolution rate in various drugs [12]. The importance of salts is highlighted by approximately 50% of the approval of the United States Food and Drug Administration (US FDA), which consists of active pharmaceutical ingredients (APIs) in salt form. Half of the top 200 prescription drugs in the US contain pharmaceutical salts [13].

It is generally accepted that the most important molecular properties for the design of a salt screen are the acid and base dissociation constants (pKa) [14]. The ΔpKa rule is also essential when designing ionic salts or neutral co-crystals. This rule states that salt formation generally occurs between an acid and a base when the ΔpKa value [pKa of the conjugate acid of the base–pKa of the acid] is greater than 3 [15,16]. Salt formation in an API largely depends on the acidity or basicity of the API itself, the safety of the salt former/counterion, choice of dosage form, route of administration, and drug indications [17]. Salt formers/counterions can be classified as cationic and anionic.

Carvedilol (CVD, (±)-[3-(9H-carbazol-4-yloxy)-2-hydroxypropyl][2-(2-methophenoxy)ethyl]amine) is an α/β adrenergic blocking agent used to treat heart failure, hypertension, and left ventricular dysfunction following myocardial infarction in clinically stable patients [18]. CVD is on the WHO Model List of Essential Medicines and represents one of the most commonly prescribed medication [19]. CVD is a BCS class II drug characterized by a high permeability; however, its oral absorption and bioavailability are limited by its low aqueous solubility [20]. Continuous efforts have been directed toward developing various techniques to improve the solubility of CVD, including particle size reduction [21], solid dispersion with excipients [22], complexation with cyclodextrin [23], and the preparation of multi-component amorphous complexes, such as co-amorphous complexes, with generally safe co-formers approved by the US FDA [24,25]. According to the Cambridge Structural Database, CVD shows good potential for forming hydrated salts with inorganic acids such as HCl, HBr, and phosphoric acid [26,27,28]. Moreover, several studies have enhanced the solubility of CVD by forming multi-component crystals with carboxylic acids such as DL-mandelic acid, benzoic acid, fumaric acid, and oxalic acid [28,29,30].

**Figure 1:**
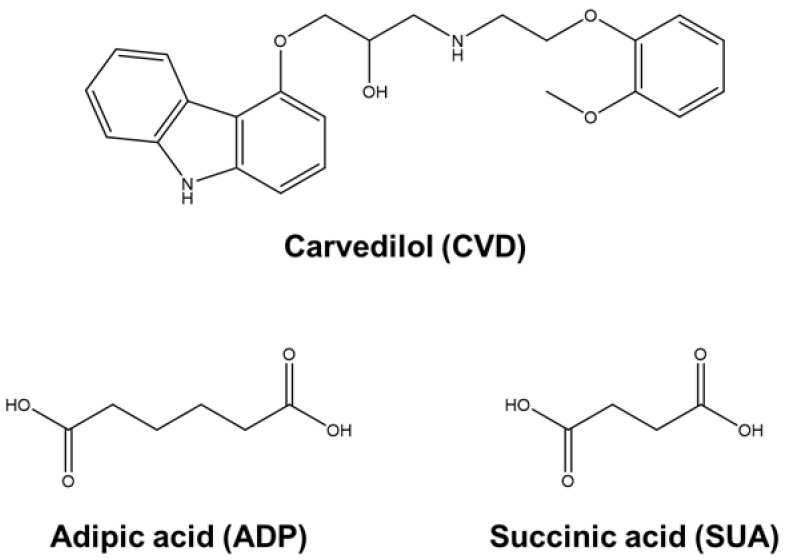
Structures of carvedilol (CVD), adipic acid (ADP), and succinic acid (SUA).

In this study, we attempted to form novel salts of CVD with dicarboxylic acids, specifically adipic acid (ADP) and succinic acid (SUA) (Figure 1), to enhance the dissolution behavior of CVD. We then investigated the physicochemical properties of the resulting salts and conducted a crystal structure analysis of the CVD salt adipate carvedilol ethanol (CVD-ADP-EtOH) and succinate carvedilol ethanol solvates (CVD-SUA-EtOH). Both compounds were characterized using powder X-ray diffraction (PXRD), Fourier transform infrared spectroscopy (FT-IR), differential scanning calorimetry (DSC), thermogravimetric (TG) analysis, and single-crystal X-ray diffraction (SCXRD) measurements. Additionally, the dissolution rate and equilibrium solubility of the salts in phosphate buffer (pH 6.8) were determined. Computational analyses were also employed to clarify the mechanism of the changes in their dissolution behavior.

## 2. Results and Discussion

### 2.1. PXRD Measurements

**Figure 2:**
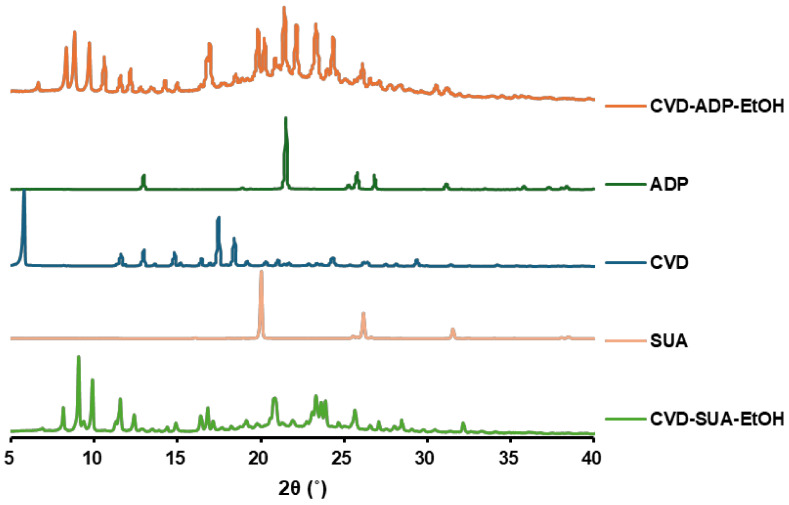
PXRD patterns of CVD-ADP-EtOH, ADP, CVD, SUA, and CVD-SUA-EtOH.

The PXRD profiles (Figure 2) were employed to confirm the presence of a novel crystalline component by comparing the patterns of CVD, SUA, and ADP with those of the newly formed samples. New PXRD patterns (2θ = 8–11° and 22–25°) were observed in CVD-ADP-EtOH and CVD-SUA-EtOH, confirming the formation of novel multi-component crystals of CVD.

### 2.2. FT-IR Spectra of the Novel Salts

**Figure 3:**
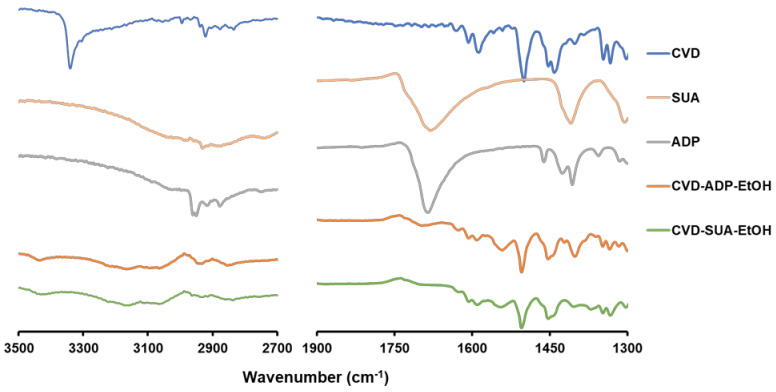
FT-IR spectra of CVD, SUA, ADP, CVD-ADP-EtOH, and CVD-SUA-EtOH.

FT-IR spectroscopy is widely employed to confirm molecular interactions in molecular complexes. The formation of a novel salt can be verified through proton transfer, and intermolecular interactions can be confirmed by detecting the shifts in the wavenumbers of the functional groups in the FT-IR spectra of the individual components and salts. In CVD-SUA-EtOH and CVD-ADP-EtOH (Figure 3), peaks at approximately 2900 cm^−1^ derived from the carboxy hydrogen in ADP and SUA disappeared. Additionally, in CVD-ADP-EtOH and CVD-SUA-EtOH, symmetric and asymmetric COO^−^ peaks were detected at wavenumbers 1540 cm^−1^ and 1405 cm^−1^, respectively. The shift from approximately 1685 cm^−1^ (C=O) observed in ADP and SUA to 1540 cm^−1^ in CVD-ADP-EtOH and CVD-SUA-EtOH corresponds to the transformation of the carboxyl group to a carboxylate group [31,32]. The absence of peaks derived from the carboxyl group observed in ADP and SUA, along with the appearance of absorptions, indicates the formation of a carboxylate anion salt. Therefore, both CVD-ADP-EtOH and CVD-SUA-EtOH are crystalline salts of CVD.

### 2.3. Thermal Properties of the Novel Salts

**Figure 4:**
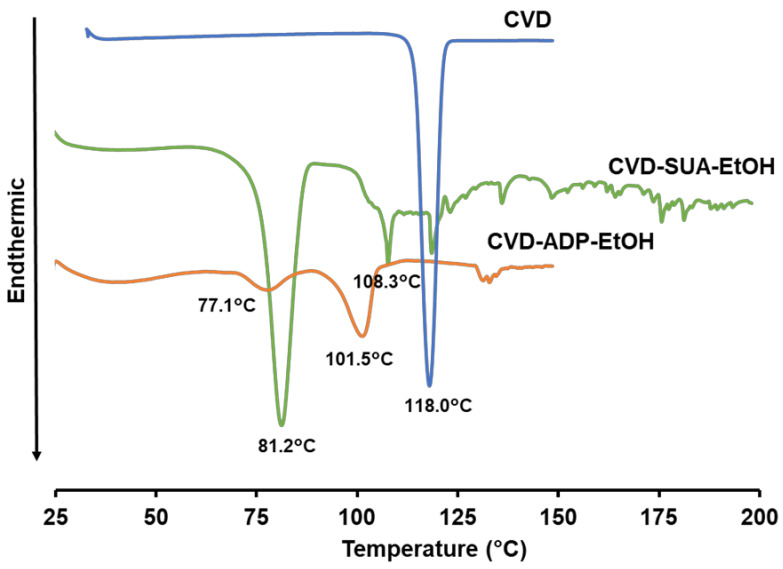
DSC profiles of CVD, CVD-ADP-EtOH, and CVE-SUA-EtOH.

**Figure 5:**
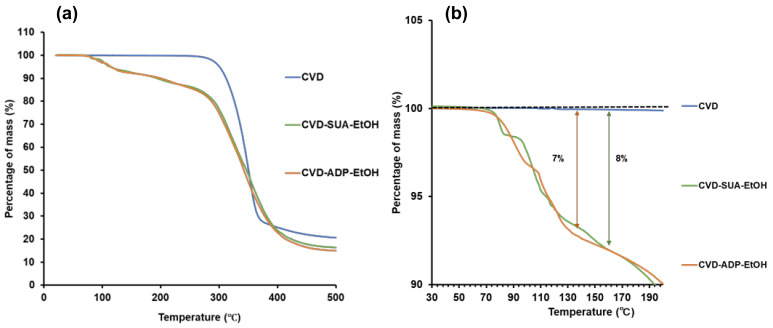
TG curves of CVD, CVD-ADP-EtOH, and CVE-SUA-EtOH (**a**) and enlarged TG figure (**b**).

The thermal properties of the two novel salts were evaluated using DSC (Figure 4), TG (Figure 5a), and an enlarged TG figure (Figure 5b). The thermogram of CVD demonstrated a sharp endothermic peak at 118.0 °C corresponding to the melting of the drug at that temperature [33]. In contrast, the DSC data revealed two endothermic peaks at 78 °C and 101.2 °C for CVD-ADP-EtOH, and two endothermic peaks at 81.2 °C and 108.3 °C for CVD-SUA-EtOH. In the TG curves of CVD-SUA-EtOH and CVD-ADP-EtOH, two distinct stages of mass loss were observed. For CVD-SUA-EtOH, an 8% mass loss occurred between approximately 80 °C and 150 °C, due to the evaporation of ethanol. Similarly, for CVD-ADP-EtOH, a 7% mass loss was observed between approximately 70 °C and 130 °C, also attributed to ethanol evaporation. These mass losses in the TG curves were consistent with the theoretical values. After the ethanol evaporation stage, degradation of each compound followed. Based on the analysis of the DSC and TG data, it can be concluded that, in both CVD-ADP-EtOH and CVD-SUA-EtOH, ethanol evaporation and compound melting may be occurring simultaneously.

### 2.4. Crystal Structures of the Novel Salts

The single-crystal structures of CVD-ADP-EtOH and CVD-SUA-EtOH were determined using SCXRD; the crystallographic information is listed in Table 1. Both CVD-SUA-EtOH and CVD-ADP-EtOH crystallized in the triclinic space group P-1, unlike pure CVD (Form II) [34], which crystallized in the monoclinic space group P21/c. Additionally, they share similarities in unit cell dimensions; however, they exhibited longer a and b lengths but a shorter c length, resulting in smaller volumes and slightly higher densities compared with pure CVD.

**Figure 6:**
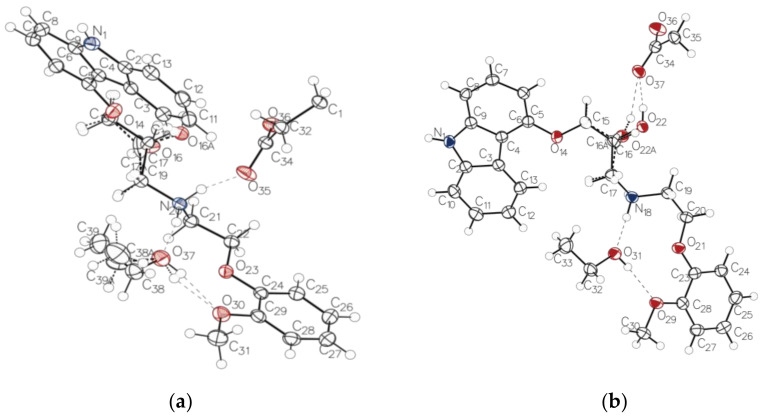
ORTEP diagram presenting the arrangement of CVD, ADP, and EtOH within the asymmetric unit of the CVD-ADP-EtOH crystal structure (**a**). ORTEP diagram presenting the arrangement of CVD, SUA, and EtOH within the asymmetric unit of the CVD-SUA-EtOH crystal structure (**b**).

The asymmetric units of CVD-ADP-EtOH (Figure 6a) and CVD-SUA-EtOH (Figure 6b) contained one CVD molecule, one ethanol molecule, and half an ADP or SUA molecule, respectively. In addition, the single-crystal structures confirmed the formation of salts with similar C-O bond lengths in the carboxylate (COO^−^) groups of CVD-ADP-EtOH (1.260 Å, 1.257 Å) and CVD-SUA-EtOH (1.255 Å, 1.257 Å). These similarities in the C-O bond lengths confirmed the transfer of an acidic proton from adipic acid and succinic acid to the aliphatic (acyclic) secondary amino group of CVD. Therefore, it can be concluded that these two novel multi-component crystals are ethanol-solvated salts of CVD.

Disorder occurred in the CVD molecule in both CVD-ADP-EtOH and CVD-SUA-EtOH. However, in CVD-ADP-EtOH, disorder also occurred in the ethanol molecule. Specifically, in CVD-ADP-EtOH, C17 and O16 of the CVD molecule were split into part 1 (C17, O16, and H16, representing a chemical occupancy of 0.741) and part 2 (C17A, O16A, and H16A, representing a chemical occupancy of 0.259). In CVD-SUA-EtOH, the C16 and O22 of the CVD molecule were split into part 1 (C16, O22, and H22, representing a chemical occupancy of 0.787) and part 2 (C16A, O22A, and H22A, representing a chemical occupancy of 0.213). Additionally, the disorder of ethanol in CVD-ADP-EtOH was split into part 1 (C38 and C39, representing a chemical occupancy of 0.915 (6)) and part 2 (C38A and C39A, representing a chemical occupancy of 0.085 (6)).

In CVD-ADP-EtOH, N20 in the CVD donates a hydrogen atom to O35 in ADP and another hydrogen atom to O37 in ethanol. Additionally, O37 in ethanol donates a hydrogen atom to O30 during CVD. The other hydrogen bond interactions in CVD-ADP-EtOH are listed in Table 2.

In CVD-SUA-EtOH, N18 donates a hydrogen atom to O36 in SUA and another hydrogen atom to O31 in ethanol. Additionally, O31 in ethanol donates a hydrogen atom to O39 of CVD. The other hydrogen bond interactions in CVD-SUA-EtOH are listed in Table 3.

**Figure 7:**
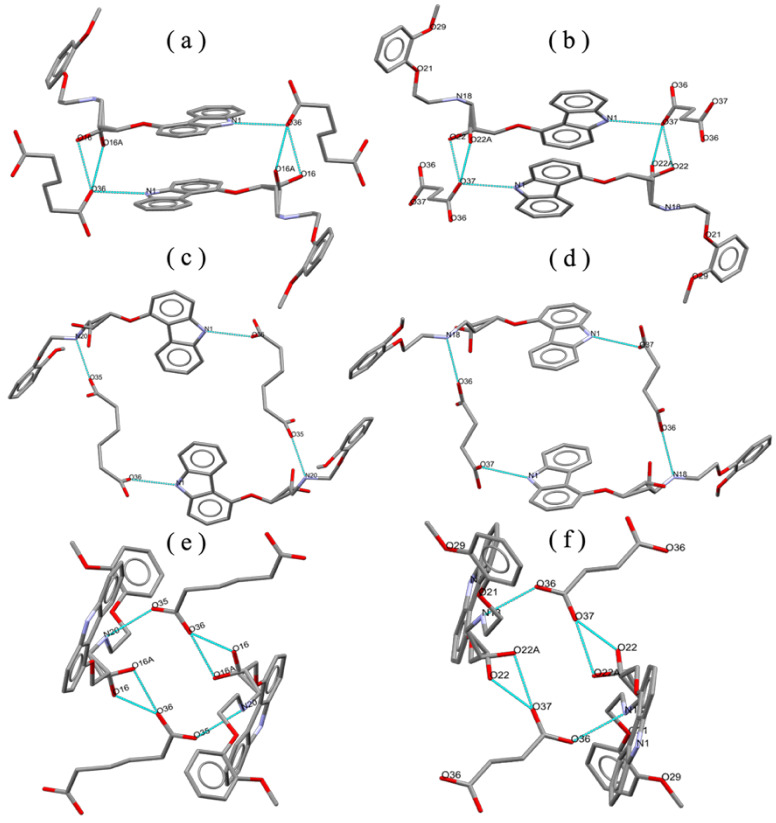
CVD is connected to ADP or SUA through hydrogen bond interactions. Three distinct types of ring structures (**a**,**c**,**e**) are formed in CVD-ADP-EtOH. Similarly, three distinct types of ring structures (**b**,**d**,**f**) are formed in CVD-SUA-EtOH.

In both CVD-ADP-EtOH and CVD-SUA-EtOH, three distinct ring structures are formed through hydrogen bond interactions between CVD and ADP or SUA, as depicted in Figure 6. An oxygen atom in ADP (O36) or SUA (O37) is connected to the nitrogen atom N1 in CVD, forming a 22-membered ring structure, as shown in Figure 7a,b. Additionally, two oxygen atoms belonging to different carboxylate anions in ADP (O35 and O36) or SUA (O36 and O37) were connected with the nitrogen atoms N20 (N18) and N1 in CVD. Because of the different number of carbon atoms in ADP and SUA, a 38-membered ring structure (Figure 7c) was formed in CVD-ADP-EtOH, while a 34-membered ring structure (Figure 7d) was formed in CVD-SUA-EtOH. Finally, the two oxygen atoms in ADP (O35 and O36) or SUA (O36 and O37), belonging to the same carboxylate anion, connect with the nitrogen atom N20 (N18) in CVD, forming 18-membered ring structures, as shown in Figure 7e,f.

In CVD-ADP-EtOH, two chains with different directions (Figures S1 and S2) were formed by the CVD and ADP molecules through hydrogen bond interactions. Similarly, in CVD-SUA-EtOH, the CVD molecules and SUA molecules are connected through hydrogen bond interactions, forming two different directional chain structures (Figures S3 and S4).

Through the analysis of their ring and chain structures, it can be concluded that CVD-ADP-EtOH and CVD-SUA-EtOH exhibit structural similarities. Despite differences in the numbers of carbon atoms between the salt formers ADP and SUA, similar molecular arrangements and intermolecular interactions between CVD and ADP and CVD and SUA accounted for the similarities in the ring structures, chain structures, and packing structures of CVD-SUA-EtOH and CVD-ADP-EtOH.

### 2.5. Dissolution Test

**Figure 8:**
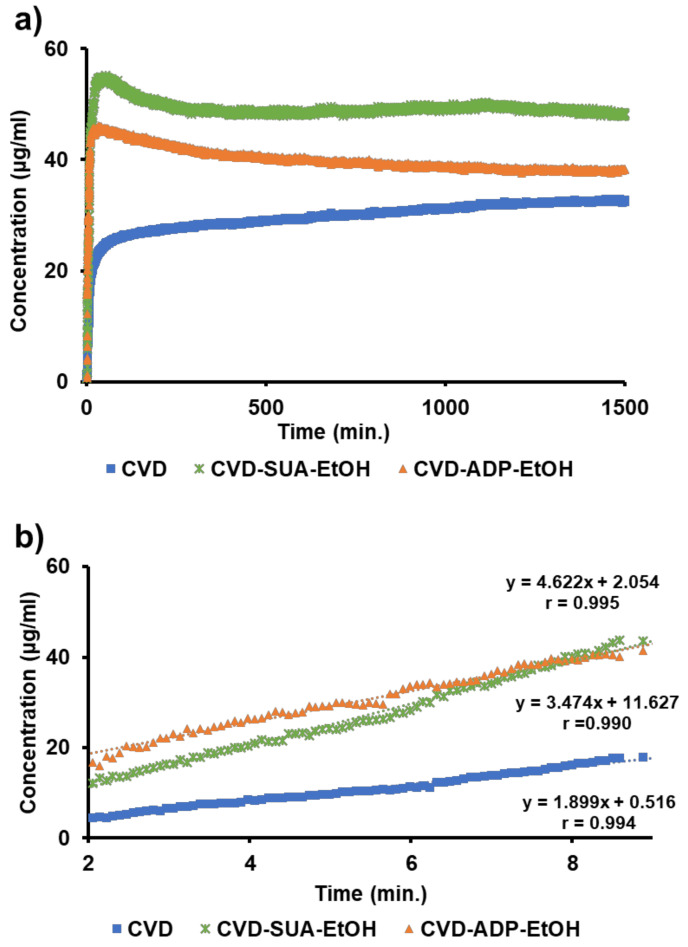
Dissolution curves of CVD, CVD-ADP-EtOH, and CVD-SUA-EtOH in a phosphate buffer (pH 6.8) were observed over 1400 min (**a**). Additionally, the dissolution curves were enlarged for the time interval between 2 and 9 min (**b**).

The average dissolution curves in a pH 6.8 phosphate buffer are shown in Figure 8, where the vertical axis represents the CVD concentration. To calculate the dissolution rate, selecting an appropriate time range was necessary. Considering the removal of air bubbles and the time delay of each sample, data from 2 to 9 min were selected to calculate the dissolution rate. The slopes of the dissolution curves indicate the dissolution rate, and the dissolution rates of CVD in CVD-ADP-EtOH and CVD-SUA-EtOH are 4.62 and 3.47 µg/mL/min, respectively. In contrast, the CVD dissolution rate of the original drug was 1.90 µg/mL/min. From these results, it can be inferred that, compared with the original CVD drug, the dissolution rate of CVD can be enhanced approximately two-fold through the synthesis of CVD-ADP-EtOH and CVD-SUA-EtOH.

### 2.6. Effect of Attachment Energy on the Dissolution Rate of CVD-ADP-EtOH and CVD-SUA-EtOH

Considering the chemical occupancy values, particularly in CVD-ADP-EtOH and CVD-SUA-EtOH, part 1 significantly outweighs part 2, leading to the removal of part 2 from the CIF file because energy calculations cannot be performed accurately when there is disorder in the structure. Therefore, when we calculated the energy, we removed part 2 of the structure to eliminate disorder. Furthermore, the disorder of ethanol in CVD-ADP-EtOH was notable, with part 1 exceeding 0.9. Therefore, the disordered part 2 was removed before calculating the attachment energy.

**Figure 9:**
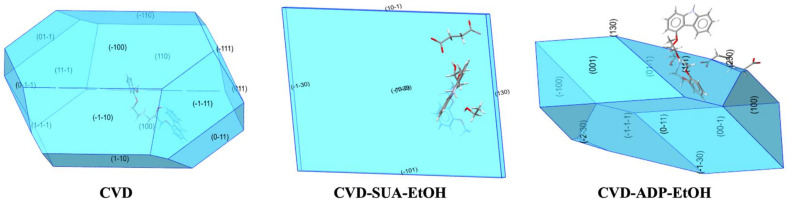
The crystal morphology of CVD, CVD-SUA-EtOH, and CVD-ADP-EtOH as calculated from their respective unit cells.

The crystal shape and surface area are known to significantly affect the dissolution rate. Therefore, the morphologies and attachment energies of CVD, CVD-SUA-EtOH, and CVD-ADP-EtOH were predicted based on the single-crystal structure units. The crystal shapes and facets of the three compounds are shown in Figure 9. Attachment energy is defined as the bond energy released when a building unit attaches to the surface of the crystal facet. The electrostatic, van der Waals, and hydrogen bond energies for each surface are listed in Table 4. Assuming that the total surface areas of the three crystals were identical, the attachment energy (E_facets_) of each crystal facet was calculated by multiplying the surface energy (E_surface_) of that specific facet by the area percentage (A%) of the entire crystal surface area occupied by the facet, as illustrated in the equation:

E_facets_ = E_surface_ × A% (1)

where E_facets_ is the attachment energy of the crystal facet, E_surface_ is the attachment energy of the surface, and A% is the area percentage the facet occupies relative to the entire crystal surface area.

The total attachment energy of the crystal molecule was then obtained by calculating the sum of the attachment energies for all facets, including the symmetry equivalents. The total attachment energies of the three compounds are listed in Table 4. The trend in the attachment energy (absolute value) shows that CVD has a higher attachment energy than CVD-ADP-EtOH or CVD-SUA-EtOH.

**Figure 10:**
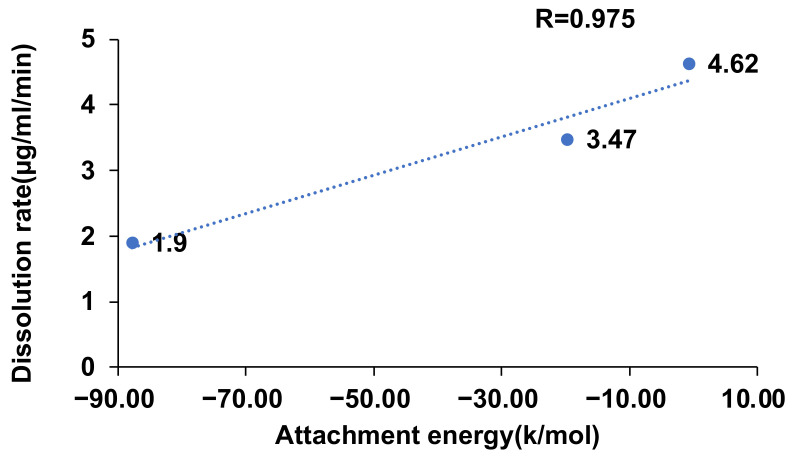
Relationship between attachment energy and powder dissolution rate.

A higher attachment energy is associated with a stronger interaction between the crystal surface and the surrounding environment. The amount of energy required to break the crystal facets decreases as the attachment energy of the crystal facet decreases. Conversely, facets with lower absolute attachment energies are considered easier to cleave, potentially resulting in lower wettability [35]. Additionally, increasing the wettability, which is associated with high cleavage, leads to a higher powder dissolution rate [36]. Therefore, it can be concluded that there is a relationship between the attachment energy and the dissolution rate, as shown in Figure 10. Based on these results, the correlation coefficient (R) between the experimental dissolution rate and the computational attachment energy value was calculated to be 0.975, indicating a strong correlation.

### 2.7. Equilibrium Solubility

**Figure 11:**
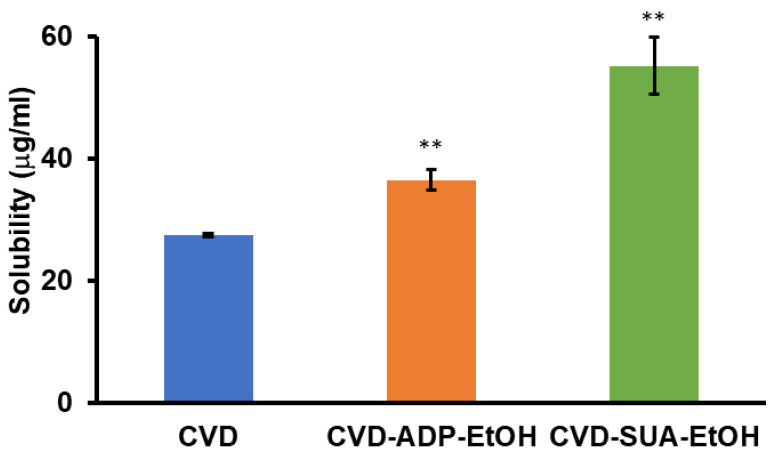
Results of the 24-h solubility tests for CVD, CVD-ADP-EtOH, and CVD-SUA-EtOH in phosphate buffer (pH 6.8) using the shake-flask method (n = 3). Student’s *t*-tests were used to determine the statistical significance of the differences with respect to CVD. ** *p* <0.01.

The 24-h solubility results for CVD, CVD-ADP-EtOH, and CVD-SUA-EtOH are shown in Figure 11. The solubility of CVD-SUA-EtOH (55.2 ± 4.7 µg/mL) is twice that of CVD (27.4 ± 0.3 µg/mL). However, the solubility of CVD-ADP-EtOH (36.5 ± 1.7 µg/mL) is only 1.3-fold higher than that of CVD. Both CVD-SUA-EtOH and CVD-ADP-EtOH showed significantly improved solubility compared with CVD.

The PXRD profiles and FT-IR spectroscopy (Figures S5 and S6) were measured after the dissolution test. When comparing the PXRD profiles with Figure 2, CVD exhibited the same PXRD patterns, indicating that no phase transition occurred during the dissolution test. However, new PXRD patterns (2θ = 9–10°, 13–14.5°, and 25–27°) in CVD-ADP-EtOH and CVD-SUA-EtOH suggest that a phase transition occurred during the dissolution process. Additionally, the identical PXRD patterns of CVD-ADP-EtOH and CVD-SUA-EtOH imply that both solvates stabilized into the same form in solution.

When comparing the FT-IR spectra with Figure 3, the symmetric and asymmetric COO^−^ peaks of CVD-SUA-EtOH and CVD-ADP-EtOH, detected at wavenumbers 1540 cm^−1^ and 1405 cm^−1^, disappeared after the dissolution test. Furthermore, typical functional group peaks for CVD were observed in both CVD-ADP-EtOH and CVD-SUA-EtOH, indicating that the insoluble powder of the two solvates after the dissolution test is composed of CVD. However, considering the possibility that phase transition may have occurred during the filtration process, the stable state of the two solvates after the dissolution test remains uncertain.

The dissolution curves (Figure 8) provide insight into the solution-mediated phase transformations of CVD-ADP-EtOH and CVD-SUA-EtOH. The dissolution profiles show that the concentration of both compounds reached a maximum at approximately 50 min before gradually decreasing. Additionally, up to 1400 min, the decrease in concentration for CVD-ADP-EtOH was greater than for CVD-SUA-EtOH. Differences in both the maximum concentration (at 50 min) and the final solubility are evident. Unfortunately, the mechanisms behind these differences remain uncertain. However, different solution-mediated phase transformations can be considered as one reason. [10,37,38].

## 3. Materials and Methods

### 3.1. Materials

CVD (Form II) and ADP were purchased from Tokyo Chemical Industry Co., Ltd. (Tokyo, Japan). Special-grade SUA was purchased from FUJIFILM Wako Pure Chemical Corporation (Osaka, Japan). All other analytical-grade solvents and reagents were commercially obtained and used without further purification. Milli-Q water was used as the purified water in all experiments. Phosphate buffer (pH 6.8) was made by mixing 0.2 mol/L disodium hydrogen phosphate solution and 0.2 mol/L sodium dihydrogen phosphate solution and adjusting the pH to 6.8.

### 3.2. Sample Preparation

CVD-ADP-EtOH and CVD-SUA-EtOH were obtained by dissolving the physical mixture of CVD and ADP or CVD and SUA (molar ratio 1:1) in ethanol, respectively, and waiting for ethanol to evaporate at 5 °C.

### 3.3. Powder X-ray Diffraction (PXRD) Measurements

PXRD measurements were taken in reflection mode using a SmartLab X-ray diffractometer (Rigaku, Tokyo, Japan) equipped with a Cu-Kα source (λ = 0.15418 Å, 45 kV, 200 mA). PXRD patterns were collected from 5° to 40° (2θ) with a step of 0.02°.

### 3.4. Fourier Transform Infrared Spectroscopy (FT-IR)

FT-IR spectra were obtained using the attenuated total reflectance (ATR) method with an FTIR-4200 spectrometer (JASCO, Tokyo, Japan) and an attenuated total reflectance unit (ATR PRO670H-S, JASCO) equipped with an internal reflection element (a diamond trapezoid with 45° entrance and exit faces). The detector used was a mercury cadmium telluride detector (MCT-4000M, JASCO). Spectra were collected from wavenumbers 400 to 4000 cm^−1^, averaging 64 scans, with a resolution of 4 cm^−1^ at 25 °C.

### 3.5. Differential Scanning Calorimetry (DSC) and Thermogravimetric (TG) Measurements

DSC and TG measurements were performed using Thermo plus EVO2-DSC 8230 and Thermo plus EVO2-TG 8120 TG-DTA instruments, respectively (Rigaku). The DSC sample (2–3 mg) was placed in a crimped aluminum pan and the TG sample (5–10 mg) was placed into an open aluminum pan. The temperature rise was 5 °C/min from 25 to 200 °C for DSC and 10 °C/min from 25 to 200 °C for TG measurements under nitrogen gas (flow rate = 50 mL/min). An empty aluminum pan was used as a reference.

### 3.6. Single-Crystal X-ray Diffraction (SCXRD) Measurements

Single-crystal XRD measurements of CVD-ADP-EtOH and CVD-SUA-EtOH were carried out in ω-scan mode with XtaLAB Synergy-i (Rigaku, Tokyo, Japan) equipped with PhotonJet-i source and an HPC area detector at 100.01 K. Diffraction data were collected and processed using CrysAlisPro 1.171.42.88a (Rigaku Oxford Diffraction, 2023). The structures of CVD-ADP-EtOH and CVD-SUA-EtOH were refined in Olex2 1.5 [39] using SHELXL 2016/6 [40]. CCDC 2320421 and 2320422 contain supplementary crystallographic data for CVD-ADP-EtOH and CVD-SUA-EtOH, respectively, which can be obtained free of charge from the Cambridge Crystallographic Data Centre at https://www.ccdc.cam.ac.uk/structures/ (accessed on 20 August 2024).

### 3.7. Dissolution Test

The dissolution test in a pH 6.8 phosphate buffer was conducted under non-sink condition using a µDISS Profiler (Pion Inc., Billerica, MA, USA) equipped with in situ fiber optic UV probes and a mini-bath platform for temperature and agitation control (Rainbow and Minibath8, MA, USA) with stirring at 37 °C (300 rpm). Approximately 5 mg of each sample was added to each vessel containing 20 mL of a pH 6.8 phosphate buffer solution. Dissolution spectra were collected every 5 s during the first 8 min, followed by spectrum collection every 30 s thereafter. The concentration of the sample and dissolution curve were analyzed using AuPro software version 7 (Pion Inc., Billerica, MA, USA). Each sample was analyzed more than three times (n ≥ 3).

### 3.8. Computational Analysis

According to the Bravais, Friedel, Donnay, and Harker (BFDH) law [41], crystal morphology could be approximated based on crystallographic geometrical considerations. In Mercury software (2023.3.0), the CSD–Particle–VisualHabit Morphology feature was utilized to calculate and visualize the attachment energy morphology [42].

The morphology of a crystal can be easily understood in terms of the interaction energy between crystalline units. To better comprehend the morphology, it was assumed that the total lattice energy (E_lattice_) was divided into the slice energy (E_slice_) and attachment energy (E_att_) as shown in the equation:

E_lattice_ = E_slice_ + E_att_ (2)

**Figure 12:**
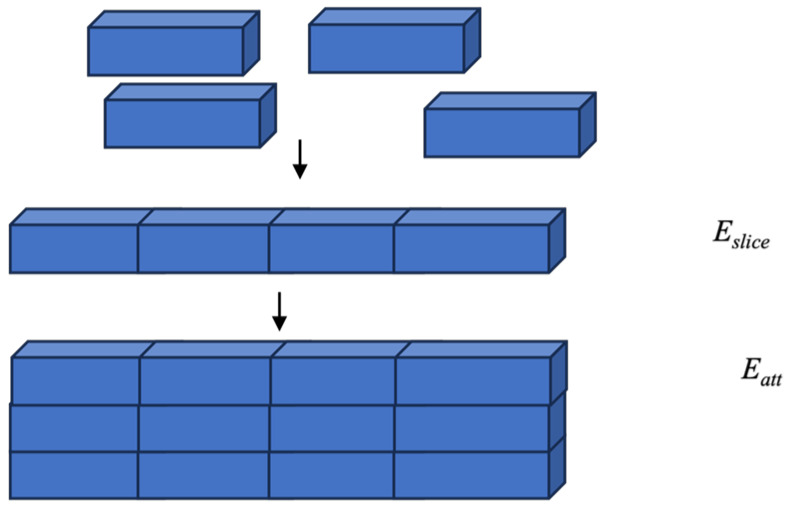
A simple schematic diagram of the definitions of E_slice_ and E_att_.

E_slice_ is defined as the energy released during the formation of a growth slice of thickness *d_hkl_*, and E_att_ is defined as the fraction of the total lattice energy released when this slice attaches to the growing crystal surface (Figure 12) [43,44,45,46,47,48,49,50].

### 3.9. Equilibrium Solubility Experiments

The solubilities of the crystals were measured using the shake-flask method, shaking 70 times/min for 24 h at 37 °C. The shaking speed was controlled using a Personal Lt-10F SX shaking water bath (TAITEC, Sapporo, Japan), and the temperature was set using an SX-10N instrument (TAITEC). Three parallel solubility determinations were conducted for each sample. After 24 h of dissolution, the solutions were filtered using a 0.45-μm filter before being subjected to HPLC. An initial 2 mL of each solution was used to saturate the filter and the subsequent filtrate was used for HPLC.

### 3.10. High-Performance Liquid Chromatography (HPLC)

The HPLC system comprised a PU-plus intelligent HPLC pump, UV-intelligent UV/VIS detector (UV-2075plus), CO-2060 plus intelligent column oven, AS-2055 plus intelligent sampler, and ChromNAV chromatography data system Ver. 1.17.01 (all from JASCO). The analytical column, an Inertsil ODS-3 (150 × 4.6 mm, particle size 5 µm; GL Sciences, Tokyo, Japan), was used at 55 °C. The mobile phase consisted of 0.05 M phosphate buffer (pH 5.0) and acetonitrile (70:30, *v*/*v*) at a flow rate of 1.0 mL/min. The injection volume of the standard sample was 10 μL, and the volume of the sample was 20 μL. The column eluate was monitored using a visible wavelength of 254 nm. A standard solution (0.1 mg/mL) was prepared using a mixture of acetonitrile and the mobile phase (1:1 ratio).

A phosphate buffer solution (pH 5.0) was prepared by dissolving 2.7 g of potassium dihydrogen phosphate in 1000 mL of water and adjusting the pH to 5.0 by adding triethylamine. Acetonitrile (428 mL) was then added to this solution, and the mixture was thoroughly blended, followed by ultrasonic deaeration.

## 4. Conclusions

In this study, crystal engineering was employed to successfully synthesize the novel multi-component systems CVD-SUA-EtOH and CVD-ADP-EtOH, and their structures were confirmed. CVD-ADP-EtOH and CVD-SUA-EtOH have similar crystal structures. This similarity arises because ADP and SUA are both dicarboxylic acids. The carboxyl groups in both SUA and ADP react with the oxygen and nitrogen atoms in the CVD within the crystal structures of CVD-ADP-EtOH and CVD-SUA-EtOH. Additionally, slight differences in the molecular structure, such as the distance between the two carboxyl groups, led to variations in the crystal structure parameters between CVD-ADP-EtOH and CVD-SUA-EtOH. Moreover, both CVD-SUA-EtOH and CVD-ADP-EtOH showed improvements in the dissolution rate and equilibrium solubility of CVD. The decreased absolute value of the attachment energy was identified as the mechanism underlying the improved dissolution rates of CVD-ADP-EtOH and CVD-SUA-EtOH. These results suggest that salt formation is an alternative method for improving CVD solubility.

## Figures and Tables

**Table 1:** Crystallographic data table for CVD (Form II), CVD-ADP-EtOH, and CVD-SUA-EtOH.

Parameters	CVD (Form II) ^(a)^	CVD-ADP-EtOH	CVD-SUA-EtOH
Empirical formula	C_24_H_26_N_2_O_4_	C_29_H_37_N_2_O_7_	C_28_H_35_N_2_O_7_
Formula weight	406.47	525.61	511.58
Temperature [K]	173(2)	100.01	100.01
Wavelength	0.71073	1.54184	1.54184
Crystal system	Monoclinic	Triclinic	Triclinic
Space group	P21/c	P-1	P-1
Unit cell dimensions			
a [Å]	15.5414 (14)	9.9404(3)	9.7393(3)
b [Å]	15.2050 (12)	10.5634(3)	10.7950(3)
c [Å]	9.1174 (8)	13.0610(3)	12.5360(3)
α [°]	90°	100.104(2)	99.058(2)
β [°]	100.730 (7)	95.155(2)	91.319(2)
γ [°]	90°	90.197(2)	90.376(2)
Volume (Å^3^)	2116.8 (3)	1344.47	1300.94
Molecules per cell (Z)	4	2	2
Molecules in the asymmetric unit (Z’)	1	1	1
Density (calculated) [g/cm^3^]	1.275	1.298	1.306
Absorption coefficient [mm⁻^1^]	0.087	0.759	0.771
F (000)	864	562	546
Crystal size [mm × mm × mm]	0.36 × 0.33 × 0.32	0.8 × 0.5 × 0.4	0.46 × 0.08 × 0.04
Theta range for data collection [°]	3.51 to 25.68	3.452 to 68.241	3.571 to 68.319
Index ranges [°]	−18 ≤ h ≤ 17, −18 ≤ k ≤ 18, −11 ≤ l ≤ 11	−11 ≤ h ≤ 11, −12 ≤ k ≤ 12, −15 ≤ l ≤ 14	−11 ≤ h ≤ 11, −12 ≤ k ≤ 12, −14 ≤ l ≤ 15
Reflections collected	3956	12,234	11,673
Independent reflections	3956	4877	4703
Completeness to theta = 67.684°	99.7%	99.7%	99.6%
µ (mm^−1^)	0.09	0.76	0.77
Absorption correction	*ω* scans	multi-scan	multi-scan
Max. and min. transmission	-	1.00000 and 0.72819	1.00000 and 0.89934
Refinement method	Fc = kFc [1 + 0.001xFc^2^λ^3^/sin(2θ)]^((−1)⁄4)	Full-matrix least-squares on F^2^	Full-matrix least-squares on F^2^
Data/restraints/parameters	3956/0/285	4877/8/386	4703/5/358
Goodness-of-fit on F^2^	1.029	1.058	1.115
Final R indices [I > 2sigma(I)]	R_1_ = 0.0403, wR_2_ = 0.0986	R_1_ = 0.0428, wR_2_ = 0.1096	R_1_ = 0.0401, wR_2_ = 0.0975
R indices (all data)	R_1_ = 0.0576, wR_2_ = 0.1056	R_1_ = 0.0455, wR_2_ = 0.1115	R_1_ = 0.0418, wR_2_ = 0.0985
Largest diff peak and hole	0.286 and −0.207	0.169 and −0.311	0.241 and −0.194
CCDC number	636656	2320421	2320422

^(a)^ Crystallographic data of CVD (Form II) is taken from the reference number [34].

**Table 2:** Geometrical parameters of the hydrogen bond interactions in CVD-ADP-EtOH.

Donor–H…Acceptor	D-H (Å)	H…A (Å)	D…A (Å)	D-H...A (°)	Symmetry Codes
N1–H1…O36	0.88	2.03	2.8841 (18)	163	x, 1 + y, z
O16–H16…O36	0.84	1.90	2.7206 (17)	164	1 − x, 1 − y, -z
N20–H20A…O23	0.91	2.53	2.8636 (16)	102	Intramolecular
N20–H20A…O37	0.91	1.92	2.8223 (17)	171	x, y, z
N20–H20B…O35	0.91	1.76	2.6372 (16)	162	x, y, z
O37–H37A…O23	0.84	2.53	3.0570 (16)	122	x, y, z
O37–H37A…O30	0.84	1.95	2.7816 (16)	169	x, y, z
C19–H19A…O14	0.99	2.44	2.8554 (19)	105	Intramolecular
C21–H21A…O16	0.99	2.42	3.092 (2)	125	Intramolecular
C21–H21B…O23	0.99	2.54	3.4637 (18)	156	1 − x, 1 − y, 1 − z
C28–H28…O35	0.95	2.42	3.3390 (19)	164	2 − x, 1 − y, 1 − z
C33–H33A…O16	0.99	2.5	3.386 (2)	149	1 − x, 1 − y, −z

**Table 3:** Geometrical parameters of the hydrogen bond interactions in CVD-SUA-EtOH.

Donor–H…Acceptor	D-H (Å)	H…A (Å)	D…A (Å)	D-H…A (°)	Symmetry Codes
CVD-SUA-EtOH					
N1–H1…O37	0.88	2	2.8430 (18)	161	1 − x, 2 − y, − z
N18–H18A…O36	0.91	1.82	2.6983 (17)	160	1 − x, 1 − y, −z
N18–H18B…O21	0.91	2.54	2.8955 (17)	104	Intramolecular
N18–H18B…O31	0.91	1.94	2.8108 (17)	160	x, y, z
O22–H22…O37	0.84	1.84	2.6671 (18)	169	x, y, z
O31–H31…O21	0.84	2.46	2.9572 (15)	119	x, y, z
O31–H31…O29	0.84	1.97	2.7937 (16)	168	x, y, z
C17–H17B…O14	0.99	2.38	2.8076 (19)	105	Intramolecular
C19–H19A…O21	0.99	2.5	3.4554 (18)	163	1 − x, 1 − y, 1 − z
C19–H19B…O22	0.99	2.41	3.025 (2)	120	Intramolecular
C27–H27…O36	0.95	2.46	3.391 (2)	168	−1 + x, y, 1 + z
C30–H30C…O22	0.98	2.58	3.238 (2)	124	1 − x, 1 − y, 1 − z

**Table 4:** Energy calculation results of each surface in CVD-ADP-EtOH, CVD-SUA-EtOH, and CVD.

	CVD-ADP-EtOH					CVD-SUA-EtOH			CVD			
Surface	(01−1)	(001)	(100)	(111)	(230)	(1−33)	(10−1)	−130	(1 0 0)	(1 1 0)	(1 1 −1)	(0 1 1)
Percentage surface area (%)	26.63	10.26	7.05	5.85	0.22	47.79	1.22	1.09	20.34	7.16	5.28	2.39
Electrostatic energy (kJ/mol)	56.09	8.53	8.65	36.81	54.26	85.59	40.03	77.21	−7.84	−7.9	−13.99	−13.9
van der Waals energy (kJ/mol)	−58.7	−21.92	−32.05	−39.97	−59.05	−61.57	−34.73	−65.86	−46.12	−75.43	−89.64	−103.61
Hydrogen bond energy (kJ/mol)	−9.39	−10.63	−12.18	−24.89	−25.04	−24.26	−15.33	−21.63	−0.01	−10.3	−19.18	−19.19
Attachment energy (kJ/mol)	−12	−24.02	−35.57	−28.05	−29.83	−0.24	−10.03	−10.28	−53.97	−93.62	−122.81	−136.69
Attachment energy of facet (kJ/mol)	−3.19	−2.46	−2.51	−1.64	−0.06	−0.11	−0.12	−0.11	−10.98	−6.7	−6.49	−3.27
Symmetry equivalent facets	(0 −1 1)	(0 0 −1)	(−1 0 0)	(−1 −1 −1)	(−2 −3 0)	(−13−3)	(−101)	(−1−30)	(−1 0 0)	(1 −1 0) (−1 1 0) (−1 −1 0)	(1 −1 −1) (−1 1 1) (−1 −1 1)	(0 1 −1) (0 −1 1) (0 −1 −1)
Total attachment energy (kJ/mol)	−19.74					−0.69			−87.78			

## Data Availability

Data will be made available on request.

## Supplementary Materials

The following supporting information can be downloaded at: https://www.mdpi.com/article/10.3390/molecules29194704/s1, Figure S1: In CVD-ADP-EtOH, the CVD molecules (green) and ADP molecules (blue) form a chain structure along the b-axis through hydrogen bonds N20--H20B…O35 and N1--H1…O36; Figure S2: The other chain is formed by hydrogen bonds N1--H1…O36 and O16--H16…O36 between CVD molecules and ADP molecules in CVD-ADP-EtOH; Figure S3: In CVD-SUA-EtOH, the CVD molecules (CVD) and SUA molecules (red) form a chain along the a-axis through hydrogen bonds N18--H18A…O36 and O22--H22…O37; Figure S4: In CVD-SUA-EtOH, the CVD molecules and SUA molecules form a chain through the hydrogen bonds O22--H22…O37 and N1--H1…O37; Figure S5: PXRD patterns of CVD, CVD-SUA-EtOH, and CVD-ADP-EtOH before and after the 24-h solubility tests; Figure S6: FT-IR spectra of CVD, CVD-SUA-EtOH, and CVD-ADP-EtOH after the 24-h solubility tests.
