# Supplementary material for: Characterization and Crystal Structural Analysis of Novel Carvedilol Adipate and Succinate Ethanol-Solvated Salts
Source: Molecules. 2024 Oct 4;29(19):4704. doi: 10.3390/molecules29194704 (PMC11478148; doi:10.3390/molecules29194704)
Supplement: Supplementary file 1 [file molecules-29-04704-s001.zip › molecules-3198022-supplementary.pdf]

molecules

Supporting Materials

Characterization and Crystal Structural Analysis of Novel Carvedilol Adipate and Succinate Ethanol-Solvated Salts

Li Ye[1], Takayuki Furuishi[2,*], Takefumi Yamashita[1] and Etsuo Yonemochi[3,*]

1 Department of Physical Chemistry, School of Pharmacy and Pharmaceutical Sciences, Hoshi University, 2-4-41 Ebara, Shinagawa-ku, Tokyo 142-8501, Japan; yorei8418@gmail.com (L.Y.), yamashita.takefumi@hoshi.ac.jp (T.Y.)
2 Juntendo University Faculty of Pharmacy,6-8-1 Hinode, Urayasu, Chiba 279-0013, Japan; t.furuishi.id@juntendo.ac.jp (T.F.)
3 School of Pharmacy at Narita, International University of Health and Welfare, 4-3, Kozunomori, Narita, Chiba, 286-8686 Japan; yone@iuhw.ac.jp (E.Y.)
* Correspondence: t.furuishi.id@juntendo.ac.jp (T.F.); yone@iuhw.ac.jp (E.Y.)

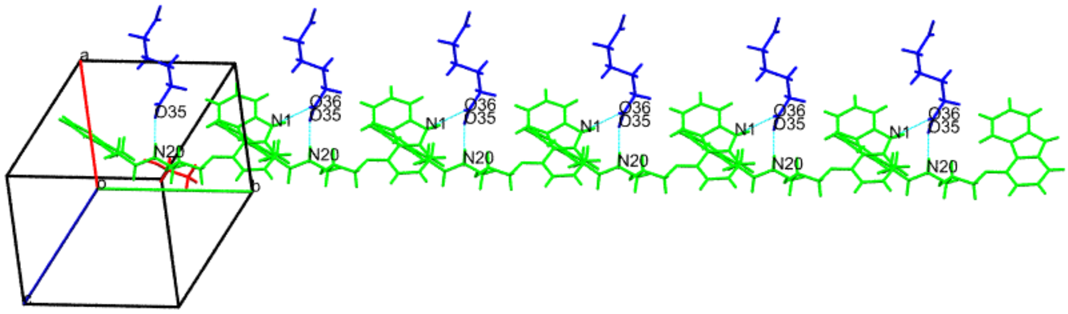

Figure S1. In CVD-ADP-EtOH, the CVD molecules (green) and ADP molecules (blue) form a chain structure along the b-axis through hydrogen bonds N20--H20B…O35 and N1--H1…O36.

Citation: To be added by editorial staff during production.

Academic Editor: Firstname Lastname

Received: date
Revised: date
Accepted: date
Published: date

Copyright: © 2024 by the authors. Submitted for possible open access publication under the terms and conditions of the Creative Commons Attribution (CC BY) license (https://creativecommons.org/licenses/by/4.0/).

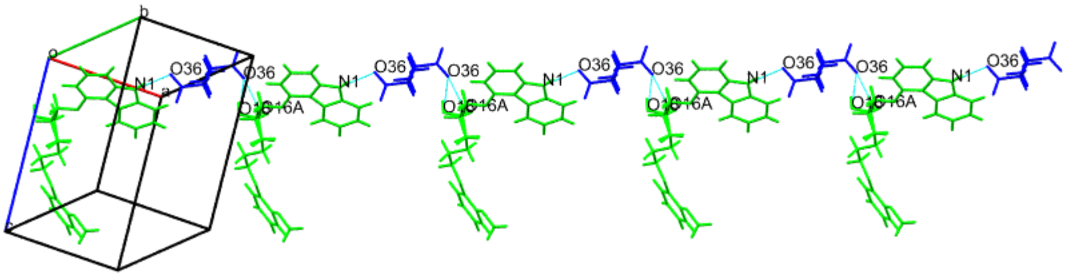

Figure S2. The other chain is formed by hydrogen bonds N1--H1…O36 and O16--H16…O36 between CVD molecules and ADP molecules in CVD-ADP-EtOH.

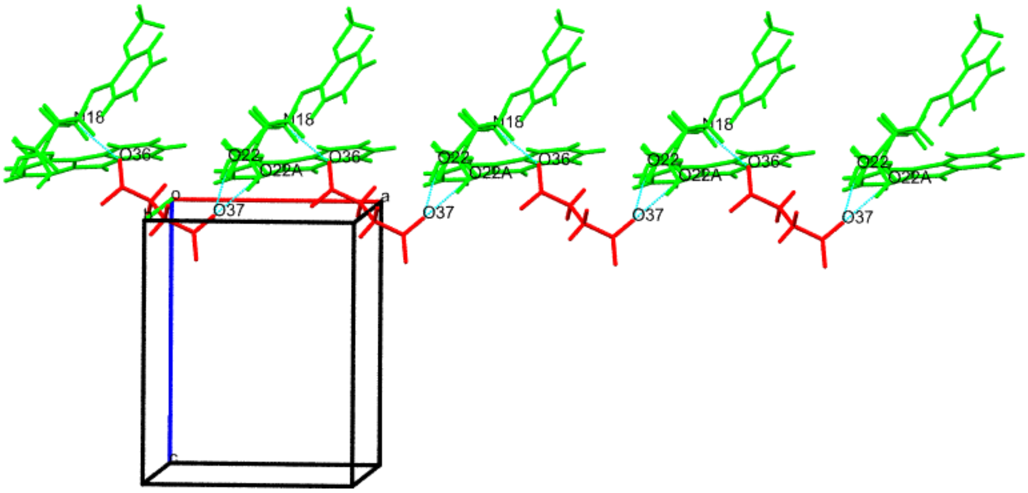

Figure S3 In CVD-SUA-EtOH, the CVD molecules (CVD) and SUA molecules (red) form a chain along the a-axis through hydrogen bonds N18--H18A…O36 and O22--H22…O37.

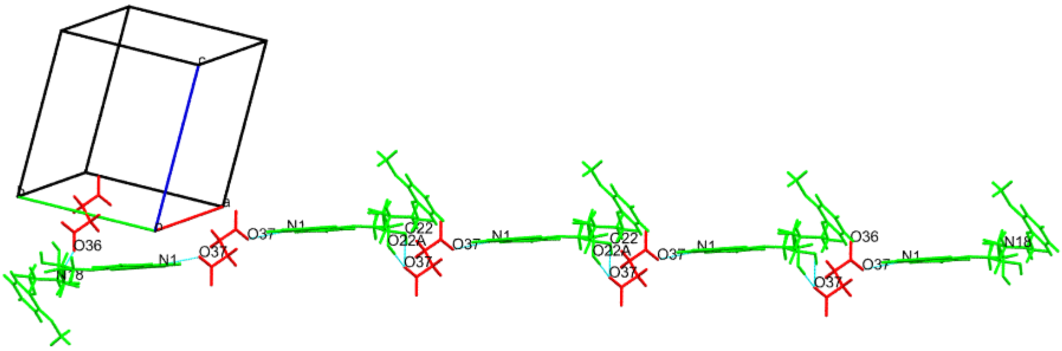

Figure S4. In CVD-SUA-EtOH, the CVD molecules and SUA molecules form a chain through the hydrogen bonds O22--H22…O37 and N1--H1…O37.

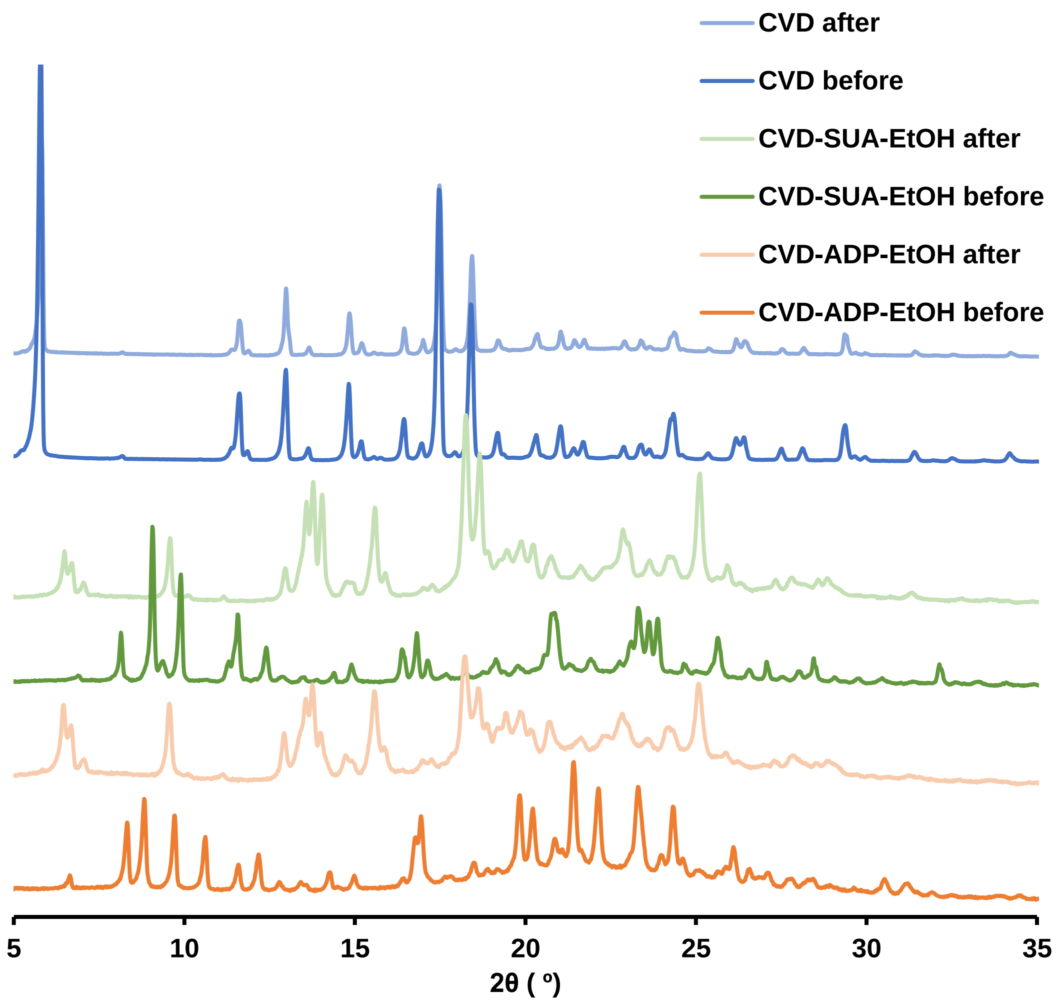

Figure S5. PXRD patterns of CVD, CVD-SUA-EtOH, and CVD-ADP-EtOH before and after the 24-hour solubility tests.

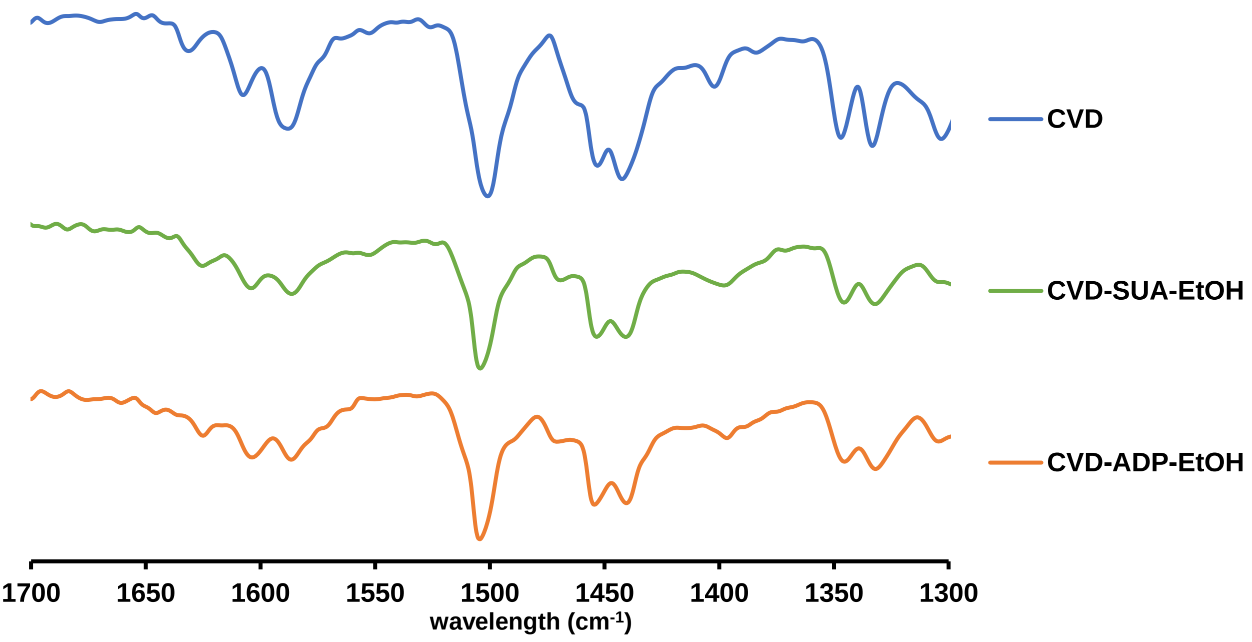

Figure S6. FT-IR spectra of CVD, CVD-SUA-EtOH, and CVD-ADP-EtOH after the 24-hour solubility tests.
